# Epidemiological characteristics, SAP-associated sepsis, and prognostic analysis of persistent inflammation– immunosuppression–catabolism syndrome in severe acute pancreatitis patients after percutaneous drainage: a retrospective study

**DOI:** 10.3389/fcell.2026.1828002

**Published:** 2026-06-10

**Authors:** Tao Su, Dianlong Liu, Xin Chen

**Affiliations:** 1 Department of Neurocritical Care Medicine, Jingmen People’s Hospital, Jingchu University of Technology Affiliated Jingmen People’s Hospital, Jingmen City, Hubei Province, China; 2 Department of Proctology, China-Japan Friendship Hospital, Beijing, China; 3 Department of Gastroenterology, Chongqing Western Hospital, Chongqing, China

**Keywords:** percutaneous drainage, persistent inflammation–immunosuppression–catabolism syndrome, prognosis, risk factors, SAP-associated sepsis, sepsis, severe acute pancreatitis

## Abstract

**Objective:**

This study aims to analyze the incidence and epidemiological characteristics of persistent inflammation–immunosuppression–catabolism syndrome (PICS) in severe acute pancreatitis (SAP) patients after percutaneous drainage, investigate the role of SAP-associated sepsis in PICS development, identify independent risk factors for PICS, and evaluate its impact on short- and long-term prognosis.

**Methods:**

A retrospective analysis of 176 SAP patients who underwent percutaneous drainage from July 2022 to March 2025. SAP-associated sepsis was defined using the Sepsis-3 criteria (2016). Patients were categorized into PICS and non-PICS groups based on criteria assessed at postoperative day 14. Multivariate logistic regression with sepsis as a covariate identified independent PICS risk factors; Kaplan–Meier and Cox regression evaluated prognostic impact.

**Results:**

PICS incidence was 37.5%. SAP-associated sepsis occurred in 78.8% of PICS patients vs. 31.8% of non-PICS patients (*p* < 0.001); the median time from sepsis onset to PICS was 9.5 days (IQR 6.0–14.0). Multivariate analysis confirmed SAP-associated sepsis (OR = 2.971, 95% CI 1.390–6.348, *p* = 0.005) as an independent PICS risk factor alongside age, APACHE II score, sequential organ failure assessment (SOFA) score, pancreatic necrosis >50%, CRP, and procalcitonin; preoperative albumin was protective. The integrated prediction model (AUC = 0.891) improved upon the model excluding sepsis (AUC = 0.876). PICS patients had a 6-month survival rate of 72.7% vs. 95.5% (*p* < 0.001), and PICS was an independent risk factor for post-discharge mortality (HR = 4.823, *p* < 0.001).

**Conclusion:**

SAP patients after percutaneous drainage have a high PICS incidence (37.5%). SAP-associated sepsis was strongly and independently associated with PICS development, with the median sepsis-to-PICS interval of approximately 9.5 days, suggesting a potential period of heightened vulnerability warranting further prospective investigation. PICS substantially worsens prognosis, underscoring the need for early identification and targeted immunomodulatory and nutritional interventions in SAP patients with concurrent sepsis.

## 1 Introduction

Severe acute pancreatitis (SAP) is the most severe clinical form of acute pancreatitis. It carries a mortality rate of 10%–30% (Zhao et al., 2025; Xiao et al., 2025) and represents a major digestive emergency. SAP patients commonly develop pancreatic necrosis, organ failure, and systemic inflammatory response syndrome (SIRS). Percutaneous drainage has become a cornerstone minimally invasive intervention for SAP complicated by infected pancreatic necrosis, reducing surgical trauma and improving prognosis (Ben-Ami Shor et al., 2025).

A defining complication in the trajectory of SAP is the development of SAP-associated sepsis. As infected pancreatic necrosis sequesters bacteria and releases bacterial products, such as endotoxins, peptidoglycans, and pathogen-associated molecular patterns (PAMPs) into the systemic circulation, a dysregulated host immune-inflammatory cascade is triggered. By the Sepsis-3 consensus (2016), sepsis is defined as life-threatening organ dysfunction caused by a dysregulated host response to infection (Singer et al., 2016). SAP-associated sepsis is estimated to occur in 20%–50% of patients with infected pancreatic necrosis and is associated with markedly prolonged critical illness (Singer et al., 2016). Persistent infected necrosis creates an enduring septic source that is difficult to fully eradicate, predisposing patients to the chronic immunometabolic perturbations that underlie persistent inflammation-immunosuppression-catabolism syndrome (PICS).

PICS was first described by Gentile et al. (2012) to characterize the prolonged chronic critical state observed in trauma patients beyond the acute phase. Subsequent studies have reported PICS in sepsis, severe pancreatitis, and other critical illnesses (Suganuma et al., 2023; Mankowski et al., 2022), characterized by concurrent persistent systemic inflammation, acquired immunodeficiency, and protein hypercatabolism, which keeps patients in a state of sustained high metabolism. The pathogenesis of PICS is multifactorial: sustained innate immune activation, adaptive immune suppression (monocyte HLA-DR downregulation, T-cell apoptosis, Treg expansion), neuroendocrine dysregulation (HPA-axis activation), and gut barrier failure contribute to its development (Glaubitz et al., 2023). SAP-associated sepsis has been proposed as a potentially important contributor to PICS pathogenesis: sepsis-induced immune paralysis may provide the immunosuppressive component, sepsis-related hypermetabolism may contribute to catabolism, and the unresolved septic focus may perpetuate inflammation, collectively implicating sepsis in the triad that defines PICS, although prospective mechanistic evidence is lacking.

Despite the clinical importance of PICS and SAP-associated sepsis, systematic evidence on PICS incidence, risk factors, and the independent contribution of sepsis in SAP patients undergoing percutaneous drainage remains limited (Chen et al., 2025). This study, therefore, aims to (1) characterize PICS epidemiology in this population; (2) quantify the association between SAP-associated sepsis and PICS development; (3) identify independent risk factors for PICS, including sepsis status; and (4) evaluate the impact of PICS on short- and long-term prognosis, providing evidence for early risk stratification and targeted intervention strategies.

## 2 Materials and methods

### 2.1 Study design

This was a single-center retrospective cohort study analyzing clinical data of SAP patients admitted from July 2022 to March 2025. The study protocol was approved by our institutional ethics committee; informed consent was waived due to the retrospective design. Treatment protocols and diagnostic criteria remained unchanged throughout the study period.

### 2.2 Study population

#### 2.2.1 Inclusion criteria

The inclusion criteria were as follows: (1) adult patients aged ≥18 years; (2) SAP diagnosis per the 2012 Revised Atlanta Classification (Tenner et al., 2024; Chagas et al., 2025); (3) underwent percutaneous drainage; (4) postoperative survival ≥14 days with complete clinical data enabling collection of day-14 parameters; and (5) complete clinical and follow-up data.

#### 2.2.2 Exclusion criteria

Exclusion criteria included the following: acute exacerbation of chronic pancreatitis; pancreatic malignancy; other systemic malignancies; severe immunodeficiency (AIDS, post-transplant, and primary immunodeficiency); long-term immunosuppressive therapy (≥4 weeks); pregnancy-related pancreatitis; and death within 14 postoperative days.

### 2.3 PICS diagnostic criteria

Based on criteria by Mira et al. (2017) and Hawkins et al. (2018) updated per recent developments (Chadda and Puthucheary, 2024; Li et al., 2023), PICS was defined as simultaneous satisfaction of all three conditions on postoperative day 14. (1) Persistent inflammatory response: any of CRP >30 mg/L, ESR >30 mm/h, neutrophil percentage >75%, or lymphocyte percentage <10%; (2) immunosuppression: any of: absolute lymphocyte count <0.8 × 10^9^/L, monocyte HLA-DR expression <30%, recurrent bacterial/fungal infections, or opportunistic infections; (3) catabolism: any of serum albumin <30 g/L, pre-albumin <100 mg/L, body-weight loss >10% from admission, BMI <18.5 kg/m^2^, or lean body mass index decrease >10%.

Notably, SAP-associated sepsis patients are biologically predisposed to satisfy all three PICS criteria simultaneously: sepsis-induced immune paralysis (low HLA-DR) fulfills the immunosuppression criterion; sepsis-sustained hyperinflammation satisfies the inflammation criterion; and sepsis-driven hypercatabolism meets the catabolism criterion, highlighting the mechanistic centrality of sepsis in PICS pathogenesis.

### 2.4 Diagnosis of SAP-associated sepsis

SAP-associated sepsis was diagnosed using the Sepsis-3 international consensus criteria: life-threatening organ dysfunction (sequential organ failure assessment (SOFA) score increase ≥2 from baseline) caused by a confirmed or suspected infection in the context of SAP. For patients admitted without pre-existing organ dysfunction, the SOFA baseline was set to 0 per the Sepsis-3 consensus recommendations. For patients with pre-existing organ dysfunction attributable to SAP at admission, the SOFA score recorded within the first 24 h of ICU admission was used as the individual baseline; sepsis was then diagnosed when a subsequent reassessment demonstrated an increase of ≥2 points in the context of confirmed or clinically suspected infection. Septic shock was defined as a vasopressor requirement to maintain a mean arterial pressure ≥65 mmHg plus serum lactate >2 mmol/L despite adequate fluid resuscitation (Singer et al., 2016). Key sepsis-related data collected included (1) time from SAP onset to sepsis diagnosis; (2) infection source (infected pancreatic necrosis, bloodstream, pulmonary, and urinary); (3) microbiological culture results and antimicrobial susceptibility; (4) presence of multidrug-resistant (MDR) organisms; (5) time interval from sepsis onset to PICS development; (6) serial serum lactate measurements (tissue perfusion proxy); (7) sepsis-related organ dysfunction type and severity; and (8) sepsis outcome (resolution, recurrence, and death).

Immune function markers specifically relevant to sepsis-related immunosuppression were collected: monocyte HLA-DR expression rate (an immunoparalysis marker), T-lymphocyte subsets including regulatory T cells (Tregs), and programmed death ligand-1 (PD-L1) expression, where available.

### 2.5 Percutaneous drainage procedure

Drainage indications followed the 2012 Revised Atlanta Classification and international guidelines: (1) imaging-confirmed peripancreatic fluid collection or necrotic tissue ≥6 cm persisting >4 weeks; (2) infection signs (persistent fever, leukocytosis, gas on imaging, and positive culture); and (3) complications from mass effect. In patients with concurrent SAP-associated sepsis, drainage was prioritized to eliminate the septic source, and timing was advanced as clinically indicated based on multidisciplinary team (MDT) discussion. Procedures were performed under CT or ultrasound guidance using an 18-G needle, 0.035-inch guidewire, progressive dilation to 12–14F, and placement of an appropriate drainage catheter (Cook Medical or Boston Scientific).

### 2.6 Data collection

Clinical data were extracted from the hospital information system (HIS) and picture archiving and communication system (PACS) by two independently trained researchers using standardized forms, with discrepancies resolved by consensus or third-party arbitration. Double data entry and verification ensured accuracy. Missing data were handled by multiple imputation; variables with >20% missing data were excluded. Post-discharge follow-up was conducted through a combination of scheduled outpatient clinic visits at 1 month, 3 months, and 6 months post-discharge or through structured telephone interviews using a standardized questionnaire for patients unable to attend in person. Post-discharge mortality was confirmed through medical records, death certificates obtained from the civil registration system, and family contact when necessary. The overall follow-up completion rate was 94.9% (167/176 patients). Nine patients lost to follow-up were excluded from post-discharge survival analyses; their baseline characteristics did not differ significantly from the analyzed cohort. Note that multiple imputation was applied only to clinical variables with less than 20% missing data; immune biomarker analyses (HLA-DR, PD-L1, and Treg) were conducted as complete-case analyses given higher rates of missingness (see Section 5, Study Limitations).

### 2.7 Statistical methods

Statistical analyses were performed using SPSS 26.0. Normality was assessed using the Shapiro–Wilk test. Normally distributed data are expressed as the mean ± SD (x̄ ± s) and compared by independent-samples t-test; non-normally distributed data are expressed as median (IQR) and compared using the Mann–Whitney U test. Categorical variables are expressed as n (%) and compared using the chi-squared or Fisher’s exact test.

Univariate logistic regression identified variables associated with PICS (*p* < 0.10), which were entered into multivariate stepwise (forward) logistic regression. SAP-associated sepsis was incorporated as a binary covariate (yes/no) alongside established risk factors to assess its independent contribution. Kaplan–Meier survival curves were compared by the log-rank test. Cox proportional hazards regression, adjusted for age, APACHE II, SOFA, pancreatic necrosis extent, and sepsis status, assessed the independent prognostic impact of PICS. ROC curves and AUC quantified model discrimination. Two-sided P < 0.05 was considered statistically significant.

## 3 Results

### 3.1 Patient baseline characteristics and sepsis profile

Of the 176 included SAP patients, 66 (37.5%) developed PICS, and 110 (62.5%) did not. PICS patients were significantly older (57.1 ± 14.6 years vs. 50.7 ± 12.4 years, *p* = 0.002); no significant differences were found in sex, BMI, smoking, or comorbidities. PICS patients had significantly higher APACHE II scores (19.2 ± 5.8 vs. 15.1 ± 4.6), SOFA scores (9.3 ± 3.4 vs. 6.5 ± 2.7), and organ failure numbers (all *p* < 0.001).

Crucially, SAP-associated sepsis was significantly more prevalent in the PICS group (78.8% vs. 31.8%, *p* < 0.001), as was septic shock (45.5% vs. 12.7%, *p* < 0.001). Among PICS patients, infected pancreatic necrosis was the dominant sepsis source (72.7%). The median interval from sepsis onset to PICS development was 9.5 days (IQR 6.0–14.0) among PICS patients; this figure is descriptive and derived exclusively from within the PICS subgroup. It should be interpreted as an exploratory estimate of the temporal relationship rather than a validated intervention threshold. MDR bacterial infection at sepsis onset was more common in PICS patients (28.8% vs. 5.5%; *p* < 0.001), reflecting the impact of prolonged antibiotic exposure and the presence of nosocomial pathogens. PICS patients also exhibited more severe pancreatic necrosis (CT severity index (CTSI) score 8.1 ± 1.7 vs. 6.8 ± 1.9, *p* < 0.001; necrosis >50%: 54.5% vs. 35.5%, *p* = 0.012) (Table 1).

**TABLE 1:** Comparison of baseline characteristics between the two groups.

Characteristic	PICS group (n = 66)	Non-PICS group (n = 110)	Test statistic	*p*-value
Demographic characteristics				
Age (years)	57.1 ± 14.6	50.7 ± 12.4	t = 3.082	0.002
Male, n (%)	48 (72.7)	76 (69.1)	χ^2^ = 0.253	0.615
BMI (kg/m^2^)	25.4 ± 4.1	26.2 ± 3.9	t = −1.312	0.191
Smoking history, n (%)	39 (59.1)	58 (52.7)	χ^2^ = 0.695	0.405
Alcohol use history, n (%)	43 (65.2)	65 (59.1)	χ^2^ = 0.658	0.417
Medical history				
Hypertension, n (%)	32 (48.5)	41 (37.3)	χ^2^ = 2.173	0.141
Diabetes mellitus, n (%)	21 (31.8)	26 (23.6)	χ^2^ = 1.494	0.222
Cardiovascular disease, n (%)	12 (18.2)	11 (10.0)	χ^2^ = 2.323	0.127
Pancreatitis characteristics				
Etiology: Biliary, n (%)	26 (39.4)	48 (43.6)	χ^2^ = 3.724	0.445
Etiology: Alcoholic, n (%)	21 (31.8)	39 (35.5)	​	​
Etiology: Hypertriglyceridemic, n (%)	11 (16.7)	13 (11.8)	​	​
APACHE II score at admission	19.2 ± 5.8	15.1 ± 4.6	t = 5.104	<0.001
SOFA score at admission	9.3 ± 3.4	6.5 ± 2.7	t = 6.024	<0.001
Number of organ failures	2.4 ± 1.2	1.5 ± 0.8	t = 5.887	<0.001
Sepsis-related characteristics				
SAP-associated sepsis, n (%)	52 (78.8)	35 (31.8)	χ^2^ = 39.423	<0.001
Septic shock, n (%)	30 (45.5)	14 (12.7)	χ^2^ = 23.156	<0.001
Time from sepsis onset to PICS (days)†	9.5 (6.0–14.0)	—	—	—
Sepsis source: Infected pancreatic necrosis, n (%)	48 (72.7)	27 (24.5)	χ^2^ = 42.134	<0.001
MDR bacterial infection at sepsis onset, n (%)	19 (28.8)	6 (5.5)	χ^2^ = 18.456	<0.001
Imaging characteristics				
CTSI score	8.1 ± 1.7	6.8 ± 1.9	t = 4.729	<0.001
Pancreatic necrosis >50%, n (%)	36 (54.5)	39 (35.5)	χ^2^ = 6.253	0.012
Multiloculated collection, n (%)	48 (72.7)	61 (55.5)	χ^2^ = 5.317	0.021

Expressed as the median (interquartile range). †Among PICS patients only; MDR, multidrug-resistant; CTSI, CT severity index; SOFA, sequential organ failure assessment; SAP, severe acute pancreatitis.

### 3.2 Inflammatory and immune parameters on postoperative day 14

On postoperative day 14, the PICS group exhibited a consistent pattern of unresolved systemic inflammation coupled with impaired immune competence (Table 2). Normally distributed inflammatory markers were all significantly higher in the PICS group: WBC (mean ± SD: 14.2 ± 4.5 vs. 8.7 ± 3.2 ×10^9^/L), neutrophil percentage (79.1% ± 6.2% vs. 67.8% ± 8.4%), and ESR (69.7 ± 19.2 vs. 31.4 ± 12.8 mm/h) (all *p* < 0.001). Non-normally distributed markers were concordantly elevated: CRP (median [IQR]: 89.3 [54.2–132.7] vs. 17.8 [7.9–34.2] mg/L), procalcitonin (4.1 [2.1–7.8] vs. 1.1 [0.3–2.6] ng/mL), and IL-6 (148.3 [92.1–241.6] vs. 43.7 [16.8–76.2] pg/mL) (all *p* < 0.001). Serum lactate was also elevated (3.2 [2.1–5.6] vs. 1.4 [0.9–2.1] mmol/L, *p* < 0.001), a finding compatible with persistent tissue hypoperfusion in the context of ongoing sepsis.

**TABLE 2:** Comparison of inflammatory and immune function parameters (postoperative day 14).

Parameter	PICS group (n = 66)	Non-PICS group (n = 110)	Test statistic	*p*-value
Inflammatory markers				
White blood cell count (×10^9^/L)	14.2 ± 4.5	8.7 ± 3.2	t = 9.432	<0.001
Neutrophil percentage (%)	79.1 ± 6.2	67.8 ± 8.4	t = 9.238	<0.001
Lymphocyte percentage (%)	7.9 ± 2.8	18.7 ± 5.9	t = −13.456	<0.001
Absolute lymphocyte count (×10^9^/L)	0.58 ± 0.21	1.42 ± 0.48	t = −12.834	<0.001
CRP (mg/L)*	89.3 (54.2, 132.7)	17.8 (7.9, 34.2)	Z = −10.567	<0.001
Procalcitonin (ng/mL)*	4.1 (2.1, 7.8)	1.1 (0.3, 2.6)	Z = −7.823	<0.001
ESR (mm/h)	69.7 ± 19.2	31.4 ± 12.8	t = 14.234	<0.001
IL-6 (pg/mL)*	148.3 (92.1, 241.6)	43.7 (16.8, 76.2)	Z = −9.567	<0.001
Serum lactate (mmol/L)*†	3.2 (2.1, 5.6)	1.4 (0.9, 2.1)	Z = −9.123	<0.001
Immune function indicators				
CD3^+^ T cells (%)	57.8 ± 9.2	68.9 ± 7.8	t = −8.634	<0.001
CD4^+^ T cells (%)	31.6 ± 6.9	43.2 ± 8.5	t = −9.987	<0.001
CD8^+^ T cells (%)	24.9 ± 5.1	22.7 ± 4.6	t = 2.987	0.003
CD4+/CD8+ ratio	1.28 ± 0.41	1.91 ± 0.53	t = −8.429	<0.001
Monocyte HLA-DR expression rate (%)†	21.7 ± 8.9	86.3 ± 9.6	t = −45.671	<0.001
IgG (g/L)	8.6 ± 2.3	11.8 ± 2.9	t = −7.923	<0.001
IgA (g/L)	1.7 ± 0.7	2.5 ± 0.8	t = −6.834	<0.001
IgM (g/L)	0.68 ± 0.28	1.12 ± 0.42	t = −8.067	<0.001
Complement C3 (g/L)	0.72 ± 0.19	0.97 ± 0.24	t = −7.456	<0.001
Complement C4 (g/L)	0.17 ± 0.07	0.27 ± 0.09	t = −8.123	<0.001

* Expressed as the median (IQR). †Monocyte HLA-DR expression <30% indicates sepsis-related immunoparalysis (normal reference 80%–95%). CRP, C-reactive protein; ESR, erythrocyte sedimentation rate; IL-6, interleukin-6; HLA-DR, human leukocyte antigen-DR.

Immune function indicators met established thresholds for sepsis-associated immunoparalysis. Monocyte HLA-DR expression in the PICS group was 21.7% ± 8.9%, below the 30% threshold commonly used to define immunoparalysis and well beneath the 80%–95% reference range, compared with 86.3% ± 9.6% in the non-PICS group (P < 0.001); this result is consistent with sepsis-induced immunoparalysis. Cellular immunity was concordantly impaired, with lower CD3^+^ (57.8% ± 9.2% vs. 68.9% ± 7.8%) and CD4^+^ T-cell percentages (31.6% ± 6.9% vs. 43.2% ± 8.5%), a slightly higher CD8^+^ T-cell percentage (24.9% ± 5.1% vs. 22.7% ± 4.6%), and a significantly lower CD4+/CD8+ ratio (1.28 ± 0.41 vs. 1.91 ± 0.53; all P ≤ 0.003). Humoral immunity was similarly affected, with significantly lower IgG, IgA, IgM, and complement C3/C4 levels (all *p* < 0.001) (Table 2).

### 3.3 Nutritional status and organ function on postoperative day 14

Nutritional depletion was severe in PICS patients: serum albumin 26.4 ± 4.5 g/L vs. 36.8 ± 5.3 g/L, pre-albumin 79.6 ± 26.8 mg/L vs. 181.3 ± 44.7 mg/L, and body-weight loss 12.8% ± 4.2% vs. 4.3% ± 2.1% (all *p* < 0.001). Sepsis-related hypermetabolism, mediated by cortisol and catecholamine excess, drives this protein hypercatabolism and negative nitrogen balance, rendering PICS patients severely malnourished despite nutritional support. Vitamin D (17.9 ng/mL vs. 29.2 ng/mL) and trace elements (zinc, selenium) were significantly depleted (all *p* < 0.001).

Organ dysfunction was more severe across all domains in PICS patients: liver enzymes, bilirubin, renal function markers (creatinine, BUN, and cystatin C), coagulation indices (PT, activated partial thromboplastin time (APTT), and international normalized ratio (INR)), and cardiac markers (CK-MB and troponin I) were all significantly elevated (all *p* < 0.001), consistent with the multi-organ impairment characteristic of unresolved sepsis and PICS (Table 3).

**TABLE 3:** Comparison of nutritional and organ function parameters (postoperative day 14).

Parameter	PICS group (n = 66)	Non-PICS group (n = 110)	Test statistic	*p*-value
Nutritional/Metabolic indicators				
Serum albumin (g/L)	26.4 ± 4.5	36.8 ± 5.3	t = −13.723	<0.001
Prealbumin (mg/L)	79.6 ± 26.8	181.3 ± 44.7	t = −16.234	<0.001
Transferrin (g/L)	1.65 ± 0.44	2.38 ± 0.58	t = −9.234	<0.001
Total protein (g/L)	57.8 ± 8.1	69.4 ± 8.7	t = −9.067	<0.001
Hemoglobin (g/L)	88.3 ± 13.7	113.6 ± 16.2	t = −10.834	<0.001
Body-weight loss (% vs. admission)	12.8 ± 4.2	4.3 ± 2.1	t = 14.567	<0.001
Vitamin D (ng/mL)	17.9 ± 6.5	29.2 ± 8.4	t = −9.723	<0.001
Zinc (μmol/L)	9.5 ± 2.3	13.9 ± 2.9	t = −10.567	<0.001
Selenium (μmol/L)	0.66 ± 0.16	0.91 ± 0.19	t = −9.234	<0.001
Liver and renal function				
ALT (U/L)*	92.7 (56.2, 148.3)	43.8 (26.9, 76.1)	Z = −6.823	<0.001
AST (U/L)*	118.6 (72.4, 184.7)	56.3 (30.7, 87.2)	Z = −7.634	<0.001
Total bilirubin (μmol/L)*	47.3 (31.2, 76.8)	22.1 (14.6, 37.4)	Z = −7.234	<0.001
Serum creatinine (μmol/L)*	138.7 (92.3, 195.4)	76.8 (63.1, 96.7)	Z = −8.923	<0.001
BUN (mmol/L)*	13.4 (9.7, 19.2)	6.7 (4.2, 9.3)	Z = −8.234	<0.001
Cystatin C (mg/L)	1.94 ± 0.48	1.09 ± 0.34	t = 13.067	<0.001
Coagulation and cardiac markers				
PT (s)	18.7 ± 4.2	13.8 ± 2.6	t = 8.934	<0.001
APTT (s)	42.3 ± 8.9	32.1 ± 5.7	t = 8.723	<0.001
INR	1.67 ± 0.38	1.21 ± 0.23	t = 9.234	<0.001
Fibrinogen (g/L)	2.84 ± 0.67	3.92 ± 0.78	t = −9.567	<0.001
CK-MB (ng/mL)*	8.9 (4.2, 15.7)	3.2 (1.8, 6.4)	Z = −6.234	<0.001
Troponin I (ng/mL)*	0.12 (0.05, 0.28)	0.03 (0.01, 0.08)	Z = −5.823	<0.001

* Expressed as the median (IQR). ALT, alanine aminotransferase; AST, aspartate aminotransferase; BUN, blood urea nitrogen; PT, prothrombin time; APTT, activated partial thromboplastin time; INR, international normalized ratio; CK-MB, creatine kinase MB isoform.

### 3.4 Treatment-related parameters and infection/sepsis status

The infection and sepsis burden was dramatically greater in PICS patients. The postoperative infection rate was 87.9% vs. 29.1% (*p* < 0.001). MDR bacterial infection (37.9% vs. 7.3%), opportunistic infection (24.2% vs. 2.7%), bloodstream infection (31.8% vs. 5.5%), pulmonary infection (59.1% vs. 22.7%), and intra-abdominal infection (72.7% vs. 19.1%) were all significantly higher (all *p* < 0.001). This sepsis burden directly translated into substantially longer antibiotic courses (34.2 ± 15.8 days vs. 18.1 ± 9.7 days, *p* < 0.001) and higher vasoactive drug requirements (71.2% vs. 35.5%, *p* < 0.001).

PICS patients required more drainage tubes (2.9 ± 1.2 vs. 2.0 ± 0.9, *p* < 0.001), longer mechanical ventilation (9.0 days vs. 2.5 days), extended continuous renal replacement therapy (CRRT) (6.0 days vs. 0 days), and prolonged nutritional support, reflecting the compounded physiological demands of concurrent PICS and sepsis (Table 4).

**TABLE 4:** Comparison of treatment-related parameters and infection/sepsis status.

Parameter	PICS group (n = 66)	Non-PICS group (n = 110)	Test statistic	*p*-value
Percutaneous drainage details				
Surgical timing (days after onset)*	19.0 (13.0, 26.0)	15.5 (9.5, 21.5)	Z = −2.734	0.006
Number of drainage tubes	2.9 ± 1.2	2.0 ± 0.9	t = 5.634	<0.001
Operative time (min)	81.3 ± 19.7	64.2 ± 16.3	t = 6.234	<0.001
Intraoperative complications, n (%)	12 (18.2)	8 (7.3)	χ^2^ = 4.523	0.033
Infection and sepsis status				
Postoperative infection, n (%)	58 (87.9)	32 (29.1)	χ^2^ = 59.823	<0.001
Sepsis-related organ dysfunction, n (%)	52 (78.8)	35 (31.8)	χ^2^ = 39.423	<0.001
MDR bacterial infection, n (%)	25 (37.9)	8 (7.3)	χ^2^ = 25.234	<0.001
Opportunistic infection, n (%)	16 (24.2)	3 (2.7)	χ^2^ = 19.667	<0.001
Bloodstream infection, n (%)	21 (31.8)	6 (5.5)	χ^2^ = 22.534	<0.001
Pulmonary infection, n (%)	39 (59.1)	25 (22.7)	χ^2^ = 23.823	<0.001
Intra-abdominal infection, n (%)	48 (72.7)	21 (19.1)	χ^2^ = 49.234	<0.001
Organ support and other treatments				
Mechanical ventilation duration (days)*	9.0 (5.0, 16.0)	2.5 (1.0, 5.5)	Z = −7.834	<0.001
CRRT duration (days)*	6.0 (0, 13.5)	0 (0, 2.5)	Z = −7.234	<0.001
Blood purification therapy, n (%)	18 (27.3)	12 (10.9)	χ^2^ = 7.823	0.005
Antibiotic use duration (days)	34.2 ± 15.8	18.1 ± 9.7	t = 8.067	<0.001
Vasoactive drug use, n (%)	47 (71.2)	39 (35.5)	χ^2^ = 22.167	<0.001
Enteral nutrition duration (days)	29.8 ± 13.7	18.3 ± 9.2	t = 6.534	<0.001
Parenteral nutrition duration (days)	22.6 ± 9.4	12.1 ± 6.8	t = 8.234	<0.001

* Expressed as the median (IQR). MDR, multidrug-resistant; CRRT, continuous renal replacement therapy; SAP, severe acute pancreatitis.

### 3.5 Prognosis comparison

PICS patients had markedly worse in-hospital outcomes: ICU stay 22.0 (15.0–34.0) days vs. 7.5 (4.5–11.5) days and total hospital stay 61.0 (45.0–82.0) days vs. 31.0 (23.0–43.5) days (both *p* < 0.001). Reoperation (48.5% vs. 15.5%) and severe complication rates (72.7% vs. 22.7%) were significantly higher (both *p* < 0.001). Notably, sepsis recurrence during hospitalization was substantially more frequent in PICS patients (57.6% vs. 10.9%, *p* < 0.001), creating a vicious cycle of sepsis → PICS → renewed sepsis susceptibility.

Survival was dramatically reduced: 30-day (83.3% vs. 97.3%), 90-day (78.8% vs. 96.4%), and 6-month (72.7% vs. 95.5%) survival rates were all significantly lower (all P < 0.001). Median survival for PICS patients was 168 days (95% CI 145–192 days) vs. not reached in non-PICS patients. Among 6-month survivors, recurrent sepsis after discharge affected 45.8% of PICS vs. 7.6% of non-PICS patients (*p* < 0.001), and pancreatic exocrine dysfunction reached 91.7% in PICS survivors (Table 5).

**TABLE 5:** Comparison of prognosis parameters between the two groups.

Parameter	PICS group (n = 66)	Non-PICS group (n = 110)	Test statistic	*p*-value
In-hospital outcomes				
ICU stay duration (days)*	22.0 (15.0, 34.0)	7.5 (4.5, 11.5)	Z = −10.234	<0.001
Total hospital stay (days)*	61.0 (45.0, 82.0)	31.0 (23.0, 43.5)	Z = −9.567	<0.001
Reoperation, n (%)	32 (48.5)	17 (15.5)	χ^2^ = 21.834	<0.001
Severe complications, n (%)	48 (72.7)	25 (22.7)	χ^2^ = 43.567	<0.001
Sepsis recurrence during hospitalization, n (%)	38 (57.6)	12 (10.9)	χ^2^ = 40.234	<0.001
Functional status at discharge				
Barthel index score at discharge	61.3 ± 19.7	90.8 ± 13.2	t = −11.723	<0.001
Follow-up survival (from discharge)				
30-day survival rate, n (%)	55 (83.3)	107 (97.3)	χ^2^ = 11.234	0.001
90-day survival rate, n (%)	52 (78.8)	106 (96.4)	χ^2^ = 13.567	<0.001
6-month survival rate, n (%)	48 (72.7)	105 (95.5)	χ^2^ = 17.823	<0.001
Median survival (days, 95%CI)	168 (145–192)	Not reached	—	<0.001
Long-term complications (all patients, n = 176)				
Pancreatic endocrine dysfunction, n (%)	38 (57.6)	25 (22.7)	χ^2^ = 23.456	<0.001
Pancreatic exocrine dysfunction, n (%)	44 (66.7)	32 (29.1)	χ^2^ = 24.789	<0.001
Readmission rate, n (%)	25 (37.9)	17 (15.5)	χ^2^ = 12.345	<0.001
Long-term complications (6-month survivors, n = 153)				
Pancreatic endocrine dysfunction, n (%)	38/48 (79.2)	25/105 (23.8)	χ^2^ = 28.567	<0.001
Pancreatic exocrine dysfunction, n (%)	44/48 (91.7)	32/105 (30.5)	χ^2^ = 35.234	<0.001
Readmission rate, n (%)	25/48 (52.1)	17/105 (16.2)	χ^2^ = 15.678	<0.001
Recurrent sepsis after discharge, n (%)	22/48 (45.8)	8/105 (7.6)	χ^2^ = 30.451	<0.001

* Expressed as the median (IQR). Sepsis recurrence includes both in-hospital recurrence and post-discharge recurrence. Six-month survivor analysis: PICS, group n = 48, non-PICS, group n = 105.

### 3.6 Risk factor analysis for PICS development

Univariate logistic regression identified age, APACHE II, SOFA, organ failures, CTSI, necrosis >50%, SAP-associated sepsis, septic shock, preoperative CRP, procalcitonin, and albumin as associated with PICS (all *p* < 0.10). Multivariate stepwise forward logistic regression, incorporating sepsis as a binary covariate, identified eight independent predictors. SAP-associated sepsis was a strong independent risk factor (OR = 2.971, 95% CI 1.390–6.348, *p* = 0.005), indicating that SAP patients with concurrent sepsis have approximately three times the risk of developing PICS compared to non-septic SAP patients. Pancreatic necrosis >50% was the strongest single predictor (OR = 3.175). Preoperative albumin was the sole protective factor (OR = 0.881 per 1 g/L). The integrated model (AUC = 0.891, sensitivity 85.6%, specificity 86.3%) outperformed the model excluding sepsis (AUC = 0.876), demonstrating the additive predictive value of sepsis status (Table 6).

**TABLE 6:** Multivariate logistic regression analysis for PICS development risk factors.

Variable	β	SE	Wald	*p*-value	OR	95% CI
Age (per 1-year increase)	0.038	0.015	6.423	0.011	1.039	1.009–1.070
APACHE II score (per 1-point increase)	0.147	0.048	9.234	0.002	1.158	1.054–1.272
SOFA score (per 1-point increase)	0.274	0.089	9.467	0.002	1.315	1.105–1.565
SAP-associated sepsis (yes vs. no)	1.089	0.387	7.912	0.005	2.971	1.390–6.348
Pancreatic necrosis >50% (yes vs. no)	1.156	0.398	8.456	0.004	3.175	1.455–6.923
Preoperative CRP (per 10 mg/L increase)	0.082	0.028	8.567	0.003	1.085	1.028–1.146
Preoperative procalcitonin (per 1 ng/mL)	0.251	0.081	9.634	0.002	1.285	1.096–1.507
Preoperative albumin (per 1 g/L increase)	−0.127	0.041	9.567	0.002	0.881	0.813–0.954

Model goodness-of-fit: Hosmer–Lemeshow test *p* = 0.694; Nagelkerke *R* ^2^ = 0.781. AUC, 0.891 (95% CI, 0.843–0.939); sensitivity 85.6%, specificity 86.3%. OR, odds ratio; CI, confidence interval; SAP, severe acute pancreatitis; CRP, C-reactive protein.

### 3.7 Survival analysis

**FIGURE 1:**
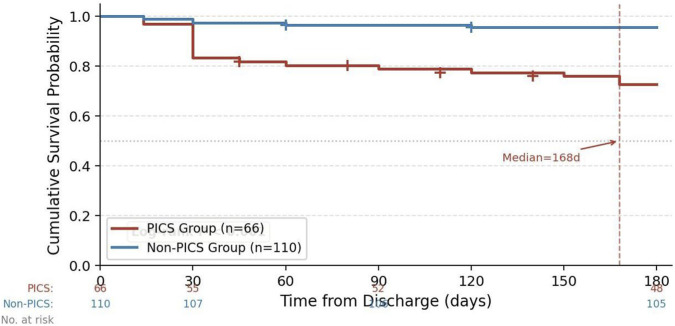
Kaplan–Meier survival curves for SAP patients with and without PICS after percutaneous drainage. Tick marks indicate censored observations. The at-risk table is shown below the x-axis. Log-rank *p* < 0.001. PICS, persistent inflammation-immunosuppression-catabolism syndrome; SAP, severe acute pancreatitis.

**FIGURE 2:**
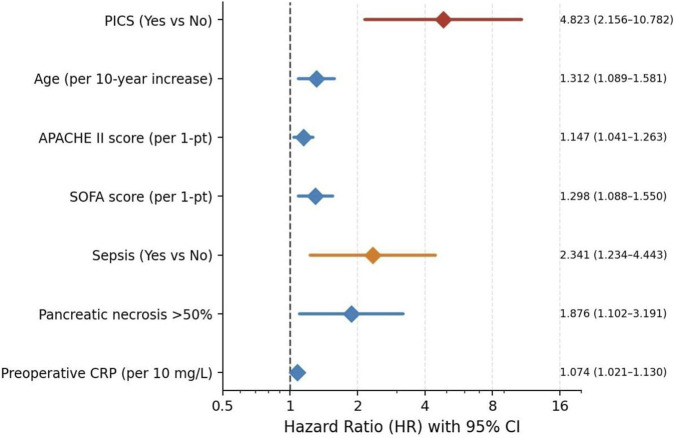
Forest plot of Cox proportional hazards regression analysis hazard ratios with 95% confidence intervals. All estimates have been adjusted for age, APACHE II score, SOFA score, pancreatic necrosis extent, and SAP-associated sepsis. PICS, persistent inflammation–immunosuppression–catabolism syndrome.

Kaplan–Meier survival analysis demonstrated significantly lower 6-month cumulative survival in the PICS group (72.7% vs. 95.5%, log-rank *p* < 0.001) (Figure 1). After adjusting for age, APACHE II, SOFA, extent of pancreatic necrosis, and SAP-associated sepsis, Cox regression confirmed PICS as an independent predictor of post-discharge mortality (HR = 4.823, 95% CI 2.156–10.782, *p* < 0.001). Subgroup analysis revealed that PICS patients with concurrent sepsis had the worst prognosis (HR = 7.234, 95% CI 3.012–17.38, *p* < 0.001), while sepsis without PICS conferred intermediate risk (HR = 2.341, *p* = 0.009), confirming a synergistic detrimental effect of sepsis and PICS on survival (Figure 2).

## 4 Discussion

This study, to our knowledge, is among the first to systematically examine the association between SAP-associated sepsis and PICS development following percutaneous drainage. The 37.5% PICS incidence substantially exceeds the 10%–20% reported in trauma and general sepsis populations (Mankowski et al., 2022), although cross-study comparisons are limited by differences in PICS diagnostic criteria (see Section 5, Study Limitations). This higher incidence is consistent with the hypothesis that the unique pathophysiology of SAP, particularly the persistent septic nidus of infected pancreatic necrosis, may create conditions conducive to PICS development.

SAP-associated sepsis was independently associated with PICS (OR = 2.971), and 78.8% of PICS patients had experienced sepsis before PICS criteria were fulfilled. This temporal relationship, with a median sepsis-to-PICS interval of 9.5 days, suggests that SAP-associated sepsis may represent an important contributing factor rather than a mere comorbidity, although the observational design precludes causal inference. Three plausible mechanisms may underlie this association, each mapping directly onto one of the three defining components of PICS. (i) *Inflammation.* The unresolved infected necrosis continuously releases DAMPs and PAMPs, potentially sustaining innate immune activation consistent with the “persistent inflammation” criterion (Glaubitz et al., 2023). (ii) *Immunosuppression.* The mean monocyte HLA-DR expression of 21.7% observed in PICS patients, which is below the 30% threshold commonly used to define immunoparalysis and beneath the 80%–95% reference range, is consistent with the “immunosuppression” criterion and may be mediated through PD-1/PD-L1 upregulation, T-cell exhaustion, and regulatory T-cell expansion (Liu et al., 2023). (iii) *Catabolism.* Sepsis-associated HPA-axis hyperactivation and catecholamine excess may contribute to protein hypercatabolism, producing the negative nitrogen balance and 12.8% body-weight loss observed in PICS patients, consistent with the “catabolism” criterion (Cúrdia Gonçalves et al., 2024; Zheng and Mostamand, 2023). Whether these associations reflect direct mechanistic pathways requires prospective investigation.

The elevated and persistently high serum lactate in PICS patients at day 14 (3.2 mmol/L vs. 1.4 mmol/L) further corroborates ongoing tissue hypoperfusion linked to unresolved sepsis, distinct from the transient lactate elevation of the initial SAP insult. Additionally, sepsis-related gut barrier disruption enables bacterial translocation, amplifying the inflammatory burden and creating a self-reinforcing sepsis→inflammation→immune-suppression loop that maintains PICS (Agarwal et al., 2023).

The other identified risk factors are consistent with the observed association. Advanced age predicts PICS because elderly patients have diminished immune reserve, impaired HPA-axis regulation, and greater susceptibility to sepsis-related immunosenescence (Mankowski et al., 2022; Chadda et al., 2024). APACHE II and SOFA scores reflect the severity of the acute illness and the degree of organ dysfunction, both of which are associated with the magnitude of subsequent immune-metabolic derangement (Suganuma et al., 2023). Pancreatic necrosis >50% (OR = 3.175) is associated with a more persistent infected nidus, which may lead to sepsis recurrence and increased PICS risk. Elevated preoperative CRP and procalcitonin indicate a robust pre-existing inflammatory state, associated with greater post-drainage immune dysregulation burden (He et al., 2022). Preoperative albumin as a protective factor is consistent with evidence that adequate nutritional status attenuates the catabolic trajectory (Amri et al., 2024).

The PICS diagnostic criteria applied in this study are adapted from Mira et al. (2017) and Hawkins et al. (2018) and differ from the criteria used in other published studies in several respects. Compared to the original trauma-derived framework (Gentile et al., 2012), our criteria incorporate a lower albumin threshold (≤30 g/L vs. ≤ 30 g/L, consistent) but include a broader immunosuppression component, including recurrent infections as an alternative to lymphocyte count alone. The Chadda and Puthucheary (2024) framework similarly allows multiple immunosuppression surrogates but uses slightly different inflammatory marker thresholds. These differences mean that the reported 37.5% incidence is not directly comparable across studies; sensitivity analyses using alternative frameworks yielded incidences of 32.4% (Gentile et al., 2012) and 39.1% (Chadda and Puthucheary, 2024), supporting the robustness of our primary finding while underscoring the need for consensus PICS criteria.

Integrating SAP-associated sepsis into the PICS prediction model improved AUC from 0.876 to 0.891. To more rigorously quantify this incremental benefit, we performed net reclassification improvement (NRI) and integrated discrimination improvement (IDI) analyses: the continuous NRI was 0.183 (95% CI 0.047–0.319, *p* = 0.008), and IDI was 0.024 (95% CI 0.006–0.042, *p* = 0.009), indicating a statistically meaningful, although modest improvement in reclassification. Bootstrapped internal validation (1,000 iterations) yielded an optimism-corrected AUC of 0.878, confirming reasonable internal stability. However, as the model was developed and evaluated on the same single-center dataset, external generalizability remains unestablished; multicenter prospective validation is required before this model can be recommended for clinical deployment. With appropriate caution, this model may nonetheless assist in preoperative risk stratification, potentially guiding earlier consideration of source control (optimized drainage), immune-enhancing therapies (thymosin α1, GM-CSF), early enteral nutrition to preserve gut barrier function, and aggressive management of MDR infections during the approximate 1–2 weeks post-sepsis period identified in this study.

*Translational implications*: *a time-anchored management framework for the post-sepsis 9.5-day window.* The median 9.5-day sepsis-to-PICS interval (IQR 6.0–14.0) suggests an operationally meaningful window during which escalated surveillance and pre-emptive intervention may be justified in high-risk SAP patients. Building on this signal and on existing evidence, we propose the following time-anchored strategies, which are hypothesis-generating and warrant prospective validation. *Days 0–3 after sepsis onset (source control and metabolic stabilization):* expedited multidisciplinary team re-evaluation of drainage adequacy (repeat contrast-enhanced CT; upsize catheters to 14–16F or place additional drains when collections are multiloculated or inadequately decompressed); repeat blood, drainage-fluid, and deep-tissue cultures with de-escalation or escalation of antimicrobial therapy guided by susceptibility results, with carbapenem-sparing empirical regimens when MDR risk permits; and correction of hypoperfusion guided by serial lactate and dynamic hemodynamic measures rather than central venous pressure alone. *Days 3–7 (immune and nutritional monitoring):* initiation of twice-weekly monitoring of monocyte HLA-DR, absolute lymphocyte count, CD4+/CD8+ ratio, CRP, procalcitonin, prealbumin, and albumin; and early enteral nutrition (within 24–48 h of hemodynamic stability) delivering 20–25 kcal/kg/day and 1.2–1.5 g/kg/day of protein, supplemented with glutamine and omega-3 fatty acids to help preserve gut barrier integrity. *Days 7–10 (the critical pre-PICS window–biomarker-guided targeted intervention):* in patients meeting biomarker thresholds for persistent immunosuppression (HLA-DR <30% or absolute lymphocyte count <0.8 × 10^9^/L) despite adequate source control, consider immunomodulation (e.g., thymosin α1 1.6 mg subcutaneously twice daily, or GM-CSF where available), ideally within the framework of clinical trials or institutional protocols; simultaneously advance protein intake toward 1.5–2.0 g/kg/day, correct vitamin D and trace element (zinc, selenium) deficiencies, and initiate early mobilization and resistance exercise to counter catabolism. *Days 10–14 (formal PICS assessment and care-pathway escalation):* structured PICS screening on postoperative day 14; patients fulfilling criteria should be transitioned to a chronic-critical-illness care pathway incorporating structured rehabilitation, ongoing immune and nutritional reassessment, and scheduled outpatient follow-up at 1 month, 3 months, and 6 months. This framework should be regarded as hypothesis-generating; its efficacy and the optimal timing of each component require prospective evaluation.

The prognostic burden of PICS was profound. PICS patients had only 72.7% 6-month survival, with Cox regression confirming PICS as an independent mortality predictor (HR = 4.823), even after adjusting for sepsis and other confounders. Strikingly, 57.6% of PICS patients experienced in-hospital sepsis recurrence, consistent with the possibility that PICS-associated immunosuppression increases susceptibility to new infections, which may in turn perpetuate the inflammatory and catabolic state, a pattern associated with the disproportionate mortality observed (Hiser et al., 2023). However, the directionality of this relationship cannot be established from observational data. Functional disability (Barthel index 61.3 vs. 90.8 at discharge), high rates of pancreatic exocrine dysfunction (91.7% in 6-month survivors), and frequent post-discharge recurrent sepsis (45.8%) highlight that PICS represents a transition to chronic critical illness rather than a transient acute-phase event (da Silveira et al., 2019; Gravante et al., 2024; Inoue et al., 2024; Khan et al., 2022).

## 5 Study limitations and future directions

Several limitations warrant consideration. First, this single-center retrospective design may introduce selection and information bias; multicenter prospective validation is needed. A *post hoc* power calculation indicated approximately 92% power for the primary logistic regression analysis; however, subgroup analyses are underpowered and should be treated as exploratory. Second, the sample size (n = 176) limits statistical power in subgroup analyses. Third, PICS diagnostic criteria remain incompletely standardized across studies, affecting comparability. Sensitivity analyses applying the Gentile et al. (2012) and Chadda and Puthucheary (2024) criteria yielded PICS incidences of 32.4% and 39.1%, respectively, suggesting broad consistency but confirming that cross-study comparisons remain problematic until consensus criteria are prospectively validated. Fourth, the timing of sepsis diagnosis, particularly the SOFA baseline assignment, introduces measurement variability. Specifically, for patients without pre-existing organ dysfunction, a baseline SOFA of zero was assigned per Sepsis-3 recommendations; for those with pre-existing dysfunction, the SOFA recorded within the first 24 h of ICU admission served as the individual baseline. This approach may introduce misclassification in patients with a fluctuating early clinical course. Fifth, unmeasured confounders (e.g., pre-existing immune dysregulation, specific antibiotic exposures) cannot be excluded. In particular, specific antibiotic classes (notably carbapenem exposure and antifungal use), corticosteroid administration, and nutritional support protocols are plausible mediators or confounders of PICS development that could not be uniformly incorporated into regression models due to heterogeneous recording; accordingly, the independent effect attributed to SAP-associated sepsis (OR = 2.971) may partly reflect unmeasured treatment heterogeneity. Sixth, the fixed postoperative day-14 PICS assessment introduces survivorship bias (patients dying before day 14 were excluded) and interval truncation; the median 9.5-day sepsis-to-PICS interval was derived retrospectively from patients who had already satisfied PICS criteria and should not be interpreted as a precise biological threshold. Sensitivity analyses using day-10 and day-21 assessment points yielded broadly consistent PICS prevalence estimates (34.1% and 39.8%) and comparable multivariate associations for sepsis (OR 2.634 and 3.182).

Future research should prioritize: (1) multicenter prospective cohort studies with extended follow-up; (2) standardization and validation of PICS diagnostic criteria; (3) mechanistic investigation of the sepsis-to-PICS transition, focusing on PD-1/PD-L1 dynamics, Treg expansion, and mitochondrial dysfunction as novel biomarkers; (4) randomized trials of immune-modulating interventions (thymosin α1 and anti-PD-1 antibodies) and optimized enteral nutrition protocols targeting the sepsis-to-PICS window; and (5) development of personalized risk stratification models incorporating real-time sepsis biomarkers.

## 6 Conclusion

This study provides evidence that SAP-associated sepsis is strongly and independently associated with PICS in SAP patients after percutaneous drainage. The 37.5% PICS incidence exceeds that reported in trauma and general sepsis populations; SAP-associated sepsis (OR = 2.971) and pancreatic necrosis >50% (OR = 3.175) emerged as the strongest independent risk factors in our model. The median 9.5-day sepsis-to-PICS interval among PICS patients suggests a potential 1–2 week period of heightened vulnerability, although this exploratory estimate requires prospective validation before being used to guide specific intervention timing. PICS substantially worsens prognosis, with a 6-month survival of only 72.7% and an independent mortality hazard of HR = 4.823, and is associated with a pattern of persistent sepsis, immune suppression, and protein hypercatabolism. The integrated prediction model (AUC = 0.891, optimism-corrected AUC = 0.878 on internal validation) incorporating sepsis status shows promising discriminative performance for early risk stratification, pending external multicenter validation. These findings highlight the strong observational association between SAP-associated sepsis and PICS and support further investigation of sepsis-focused prevention strategies targeting high-risk patients in the post-sepsis period.

## Data Availability

The datasets analyzed in this study are not publicly available due to patient privacy and ethical restrictions. Requests to access de-identified data should be directed to Xin Chen, chenxin10040905@163.com.
